# Pathology, Tissue Distribution, and Phylogenetic Characterization of Largemouth Bass Virus Isolated from a Wild Smallmouth Bass (*Micropterus dolomieu*)

**DOI:** 10.3390/v17081031

**Published:** 2025-07-23

**Authors:** Christine J. E. Haake, Thomas B. Waltzek, Chrissy D. Eckstrand, Nora Hickey, Joetta Lynn Reno, Rebecca M. Wolking, Preeyanan Sriwanayos, Jan Lovy, Elizabeth Renner, Kyle R. Taylor, Ryan Oliveira

**Affiliations:** 1 Washington Animal Disease Diagnostic Laboratory, College of Veterinary Medicine, Washington State University, 1940 Olympia Avenue, Pullman, WA 99164, USAnora.hickey@wsu.edu (N.H.); p.sriwanayos@wsu.edu (P.S.);; 2 Department of Veterinary Microbiology and Pathology, College of Veterinary Medicine, Washington State University, 1845 Ott Road, Pullman, WA 99164, USA; 3 U.S. Geological Survey, Western Fisheries Research Center, Seattle, WA 98115, USA; 4 South Dakota Game, Fish and Parks, Ft. Pierre, SD 57532, USA; elizabethrennerphd@gmail.com; 5 School of Natural Resources, University of Nebraska Lincoln, Lincoln, NE 68583, USA

**Keywords:** largemouth bass virus, iridovirus, ranavirus, smallmouth bass

## Abstract

We performed a diagnostic disease investigation on a wild smallmouth bass (*Micropterus dolomieu*) with skin ulcers that was collected from Lake Oahe, South Dakota, following reports from anglers of multiple fish with similar lesions. Gross and histologic lesions of ulcerative dermatitis, myositis, and lymphocytolysis within the spleen and kidneys were consistent with largemouth bass virus (LMBV) infection. LMBV was detected by conventional PCR in samples of a skin ulcer, and the complete genome sequence of the LMBV (99,184 bp) was determined from a virus isolate obtained from a homogenized skin sample. A maximum likelihood (ML) phylogenetic analysis based on the major capsid protein (MCP) gene alignment supported the LMBV isolate (LMBV-SD-2023) as a member of the species *Ranavirus micropterus1*, branching within the subclade of LMBV isolates recovered from North American largemouth (*Micropterus salmoides*) and smallmouth bass. This is the first detection of LMBV in wild smallmouth bass from South Dakota. The ultrastructure of the LMBV isolate exhibited the expected icosahedral shape of virions budding from cellular membranes. Viral nucleic acid in infected cells was visualized via in situ hybridization (ISH) within dermal granulomas, localized predominantly at the margin of epithelioid macrophages and central necrosis. Further sampling is needed to determine the geographic distribution, affected populations, and evolutionary relationship between isolates of LMBV.

## 1. Introduction

A globally emerging pathogen, particularly in North America and Southeast Asia, including China and Thailand, largemouth bass virus (LMBV) is a double-stranded DNA virus belonging to the *Ranavirus* genus of the *Iridoviridae* family [1]. Like other ranaviruses, LMBV has a broad host range, is capable of infecting multiple species, and is most notable for causing high mortality and great economic losses in numerous species of Centrarchidae, including largemouth bass (*Micropterus salmoides*), bluegill (*Lepomis macrochirus*), and, to a lesser degree, smallmouth bass (*Micropterus dolomieu*) [2,3,4,5,6].

Both largemouth bass and smallmouth bass are economically important species within the freshwater aquaculture and sport fishing industries. Largemouth bass are intensively cultured in many countries, with rapid increases in the last decade, and currently represent the seventh largest freshwater aquaculture species in China, with a total annual production of 802,486 tons in 2022, based on data from the China Fisheries Statistical Yearbook [7]. In North America, bass are prized and highly sought-after sport fish. In the United States, black bass, or fish of the *Micropterus* genus, which includes both largemouth bass and smallmouth bass, is the most pursued category of freshwater fish, based on both numbers of anglers and days of fishing. In 2001, an estimated USD 116.1 billion in US economic output was generated by 34.1 billion anglers, of which 10.7 million (38% of the freshwater anglers) were bass fishermen [8]. Smallmouth bass are also ecologically important as a top predator in freshwater ecosystems, regulating prey populations of insects, crayfish, and smaller fish, and influencing the overall balance of the food web [9]. Black bass are considered to have a high thermal tolerance, with juvenile fish able to tolerate warmer water temperatures than adults [10]. Temperature was found to be a primary factor in smallmouth bass habitat selection in spring-fed Missouri streams, with smallmouth bass younger than one year old selecting warmer available habitats [11].

Typically, LMBV-infected fish exhibit a wide variety of clinical signs and lesions, including skin ulcers, lethargy, erratic swimming due to swim bladder overinflation, erythematous or darkened skin pigmentation, exophthalmos, internal and external hemorrhages, and organomegaly. These correspond to histopathologic lesions of multifocal necrosis and hemorrhage within the liver, spleen, kidneys, skeletal muscle, and visceral fat along with lymphocytolysis in the spleen and kidneys [2,12,13].

Given the broad host range and the potential to cause high mortality in multiple species of economic importance, development of tools for characterization of LMBV infection in smallmouth bass is critical to better understand how this virus causes disease, economic losses, and population declines in both wild and managed populations of fish.

In October 2023, one adult smallmouth bass with multifocal skin ulcers was collected by angling from Lake Oahe by the South Dakota Department of Game, Fish and Parks and submitted for diagnostic evaluation to the Washington Animal Disease Diagnostic Laboratory (WADDL). Starting in mid-September, multiple fish with similar ulcerative lesions were reported by alarmed anglers from Lake Oahe, an area with high sport fishing pressure, with large numbers of fish collected and released. Several walleye (*Sander vitreus*) close to the state record in size were caught in the area within the preceding month, leading to a major spike in fishing pressure in September and October, and a gillnetting survey had been performed from September 3rd to the 27th by the South Dakota Department of Game, Fish and Parks [14]. In this study, we report the clinical, gross, and histopathologic findings from the infected smallmouth bass, ancillary diagnostics, isolation and sequencing of LMBV, tissue localization of viral nucleic acid via in situ hybridization (ISH), virion visualization using transmission electron microscopy, and phylogenetic analysis of the virus based on the major capsid protein gene.

## 2. Materials and Methods

### 2.1. Sample Collection

In October 2023, after receiving reports from concerned anglers about fish observed with skin lesions, one adult smallmouth bass weighing 1696 g and measuring 45 cm in length was collected from the Swan Creek region of Lake Oahe by the South Dakota Department of Game, Fish and Parks. The fish was caught by angling, humanely euthanized using tricaine methanesulfonate (MS-222), and submitted whole, on ice, for diagnostic evaluation to the Washington Animal Disease Diagnostic Laboratory (WADDL). A routine necropsy was performed. A skin sample from an ulcerative lesion was collected and stored in a sterile Whirl-Pak^®^ (Pleasant Prairie, WI, USA) bag. Spleen and kidney were pooled in a sterile Whirl-Pak^®^ bag for virus isolation. Samples of fresh brain and kidney were sampled aseptically for inoculation on Columbia blood agar (CBA) and tryptone yeast extract (TYE) agar plates and incubated at 20 °C for 7 days. The resulting colonies were identified using Gram staining, catalase biochemical tests, and a matrix-assisted laser desorption time-of-flight mass spectrometer (MALDI-TOF MS, Sirius, Bruker, Billerica, MA, USA) following the manufacturer’s extended direct transfer protocol. Wet mounts of skin and gill biopsies were evaluated via light microscopy for organisms including external parasites, fungi, oomycetes, or bacteria. Tissues collected at the time of necropsy and examined histologically include the skin, anterior and posterior kidney, liver, spleen, heart, gills, ovary, eye, and brain, among others. Samples of these tissues were fixed in 10% neutral buffered formalin.

### 2.2. Histopathology

Formalin-fixed tissues collected at necropsy were routinely processed and embedded in paraffin blocks. Subsequently, 5 μm sections were cut, placed on glass slides, and stained with hematoxylin and eosin (H&E) for examination using light microscopy with a Nikon Eclipse 50i microscope. Slides were subsequently digitized using a Leica GT450 scanner for capture of digital images.

### 2.3. In Situ Hybridization

In situ hybridization (ISH; RNAscope, Advanced Cell Diagnostics (ACD)) was used to visualize viral nucleic acid in formalin-fixed skin, spleen, and kidney tissue embedded in paraffin wax. A set of antisense-specific RNA probes comprised of 20 Z pairs targeting nucleotides 257–1322 of the LMBV major capsid protein gene (Gen Bank FR682503.1) was developed by ACD. The ISH assay was performed according to manufacturer’s protocols with the following specific conditions. Formalin fixed, paraffin-embedded (FFPE) tissues were sectioned at 4 µm on charged slides. Samples were submerged in target retrieval solution for 15  min, followed by incubation with RNAscope^®^ Protease Plus (ACD, Newark, CA, USA) at 40 °C for 30 min. A positive control probe for smallmouth bass *cytochrome b* (GenBank NC_011361.1) and a negative control probe for the *dapB* gene of *Bacillus subtilis* (GenBank EF_191515) were applied to test and control tissues. Slides were examined by light microscopy with a Nikon Eclipse 50 i microscope (Nikon, Tokyo, Japan). Slides were subsequently digitized using a Leica GT450 scanner (Leica Biosystems Imaging, Inc., Vista, CA, USA) for capture of digital images.

### 2.4. Nucleic Acid Extraction and PCR Amplification

The DNA from a fresh skin ulcer, FFPE skin ulcer, and FFPE spleen tissue was extracted from the fish using Qiagen^®^ QIAamp^®^ DNA Mini Extraction Kit (QIAGEN, Venlo, The Netherlands), according to the manufacturer’s instructions, with the following modifications: 40 µL Proteinase K added to lysate for fresh tissues and as previously described for FFPE tissues [15]. A LMBV conventional polymerase chain reaction (PCR) assay targeting a portion of the major capsid protein (MCP) gene was performed as published [16] with the following modifications: thermal cycling conditions were set at 95 °C for 3 min 15 s, followed by 35 cycles of 95 °C for 45 s, 60 °C for 45 s, and 72 °C for 1 min, with a final extension of 72 °C for 7 min; hold at 4° C. The 248 bp PCR product was visualized on a 1.5% agarose gel, purified using a Quantum Prep Freeze ‘N Squeeze™ DNA Gel Extraction Spin Column (BIO-RAD, Hercules, CA, USA), and sequenced in both directions at an outside facility (Azenta, South Plainfield, NJ, USA). Forward and reverse sequences were aligned using MUSCLE 5.1 and the consensus sequence was analyzed using BLASTN v2.16.0+ [17].

### 2.5. Virus Isolation

Virus isolation was attempted on *Epithelioma papulosum cyprini* cells (EPC) incubated at 15 °C and maintained in methylcelluose (MC, Sigma-Aldrich, St. Louis, MO, USA), supplemented with 5% fetal bovine serum (FBS, Avantor, VWR, Caseyville, IL, USA), 1% PenStrep (PS, Sigma-Aldrich, St. Louis, MO, USA), and 1% GlutaMax (Glut, Gibco, Life Technologies, Grand Island, NY, USA); Chinook salmon embryo cells (CHSE-214) incubated at 15 °C and maintained in RPMI-1640 (RPMI, Sigma-Aldrich, St. Louis, MO, USA) supplemented with 5% fetal bovine serum, 1% PenStrep, 0.1% Gentamicin (Gent, Sigma-Aldrich, St. Louis, MO, USA), and 0.5% Amphotericin B (Fungizone, Sigma-Aldrich, St. Louis, MO, USA); bluegill fry cells (BF-2) incubated at 15 °C and maintained in minimum essential medium supplemented with Earle’s salts, (MEM-Earles, Sigma-Aldrich, St. Louis, MO, USA) supplemented with 5% fetal bovine serum, 1% PenStrep, and 0.1% Gentamicin; and fathead minnow cells (FHM) incubated at 15 °C and 25 °C and maintained in minimum essential medium supplemented with Hank’s salts (MEM-Hanks, Sigma-Aldrich, St. Louis, MO, USA), supplemented with 5% fetal bovine serum, 1% PenStrep, 0.1% Gentamicin, and 0.5% Amphotericin B.

A sample of pooled kidney and spleen was weighed and diluted 1:1 in calcium magnesium free salts buffer solution (Thermo Fisher Scientific, Hanover Park, IL, USA) prior to manual homogenization. After being transferred to a clean tube, the homogenate was clarified by centrifuging at 4 °C at 2000× *g* for 15 min. The supernatant was then inoculated at a 1:20 dilution to another clean tube containing processing medium RPMI-1640, supplemented with 5% fetal bovine serum, 1% PenStrep, 0.1% gentamicin, and 0.5% Amphotericin B. The sample was allowed to incubate overnight at 4 °C prior to repeated clarification as described above. Then, 50 µL polyethylene glycol (PEG, Thermo Fisher Scientific, Hanover Park, IL, USA) was added to wells for the EPC cell line prior to inoculation. Following this, 100 µL of the supernatant was inoculated at further dilutions of 1:10, 1:100, and 1:1000 onto a confluent monolayer of each cell line into 2 wells for each dilution of a 24-well plate (Thermo Fisher Scientific, Hanover Park, IL, USA). Two separate wells were inoculated with 100 µL of processing medium as a negative control per cell line. The 24 well plates were placed on a rocker (Thermo Fisher Scientific, Hanover Park, IL, USA) for one hour at 15 °C, after which all cell lines were fed with 1 mL of appropriate feed media and incubated at 15 °C. The FHM cell line was also incubated at 25 °C.

After 14 days of incubation, a single well of the 1:10 dilution for each sample was scraped, harvested, and incubated overnight at 4 °C. Prior to inoculating onto new cells, the harvested material that was incubated overnight was centrifuged at 4 °C at 2000× *g* for 15 min. The harvested cell suspension was then inoculated onto new cells as described above for initial sample inoculation. The cells were observed three times per week for cytopathic effects (CPE).

### 2.6. Whole-Genome Sequencing and Phylogenetic and Genetic Analyses

Spent cell culture supernatant was collected following the observation of CPE in the first passage of the FHM cell line five days post inoculation for purification of total viral DNA using a QIAamp DNA Micro kit (Qiagen, Hilden, Germany) according to the manufacturer’s instructions. A sequencing library was prepared using a DNA Prep kit (Illumina, San Diego, CA, USA) and sequenced on a NextSeq 1000 instrument (Illumina, San Diego, CA, USA) using a P1 600-cycle reagent kit. Data were analyzed using default options in CLC Genomics Workbench, version 24.0 (https://digitalinsights.qiagen.com, (accessed on 19 May 2025)) unless otherwise noted. Reads were trimmed and host reads (i.e., FHM cell line) were removed by aligning the trimmed reads against a fathead minnow (*Pimephales promelas*) genome assembly (RefSeq assembly GCF_016745375.1). De novo assembly of the unmapped reads was performed using SPAdes v3.14.1 [18] with the --careful, --cov-cutoff auto, and -k 33,77,127 options. Contigs related to the genomes of members of the family *Iridoviridae* were identified by using BLASTX v2.14.1+ analysis [19] against the NCBI non-redundant protein database. The integrity of the resulting iridoviral contigs was verified by visually inspecting the alignment of the unmapped reads to the contigs in QIAGEN CLC Genomics Workbench, version 24.0 (https://digitalinsights.qiagen.com, (accessed on 19 May 2025)). Open reading frames (ORF) of the assembled genome (LMBV-SD-2023 isolate) were predicted using the Genome Annotation Transfer Utility (https://4virology.net/virology-ca-tools/gatu/, (accessed on 24 May 2025)), with LMBV-WVL21117 (GenBank accession no. PP526145) used as the reference genome.

The major capsid protein (MCP) gene sequences were retrieved from 54 ranaviruses available in the NCBI GenBank database including the LMBV isolate (SD-2023) sequenced in the current study (Table S1). Nucleotide gene alignments were generated using MUSCLE within CLC Genomics Workbench. A maximum likelihood (ML) analysis was performed using IQ-TREE v1.6.12 (http://iqtree.cibiv.univie.ac.at/, (accessed on 20 May 2025)) with the auto feature to determine the best model fit and 1000 non-parametric bootstraps to test the robustness of the clades.

### 2.7. Electron Microscopy

One nearly-confluent 80 cm^2^ cell-culture treated flask (Thermo Fisher Scientific, Cat. number 12-565-50) of FHM cells was inoculated with 25 μL of second-passage cell lysate. The cells were fixed 2 days post-inoculation (dpi) in 15 mL of modified Karnovsky’s fixative (2P + 2G, 2% formaldehyde prepared from paraformaldehyde and 2% glutaraldehyde in 0.1 M cacodylate buffer pH 7.4) at room temperature for 2 h, followed by scraping the cells into a 15 mL conical tube that was stored at 4 °C until shipping to the U.S. Geological Survey Western Fisheries Research Center. The cell pellet was washed twice in cacodylate buffer for 10 min each. Washing was conducted by centrifugation at 3260× *g* for 10 min in a Sorvall ST 8R centrifuge (Thermo Fisher Scientific) to pellet the cells to decant the solution, followed by resuspension in fresh buffer. Secondary fixation of the cell pellet was conducted at room temperature for 1 h in 1% osmium tetroxide (Electron Microscopy Sciences, Hatfield, PA, USA, Cat. # 19190) diluted in cacodylate buffer. Cells were again washed with two changes in cacodylate buffer, each for 10 min, followed by centrifugation to pellet the cells. The buffer was decanted and replaced with warm 1% agarose (Sigma, Cat. #A9539) and allowed to solidify at −20 °C for 10 min. The agarose-embedded pellet was removed from the centrifuge tube and cut into 2 mm pieces for further processing. Samples were dehydrated through an ascending series of ethanols, including two changes of each solution of 50%, 70%, and 95% ethanol for 10 min each, followed by two changes of anhydrous ethanol for 15 min each. Samples were cleared with two changes of acetone for 10 min each. Infiltration with EMBed-812 resin (Electron Microscopy Sciences, Cat. # 14121) was conducted at room temperature in a 1:1 ratio of resin to acetone for 2 h, followed by a 3:1 ratio of resin to acetone for 2 h, and finally into pure resin maintained overnight in a vacuum desiccator. The samples were embedded in BEEM capsules (size 00, Ted Pella, Redding, CA, USA, Cat. #130–1) in pure resin and polymerized in a vacuum oven at 60 °C overnight. Sectioning was conducted using a Leica Ultracut UCT ultramicrotome. Semi-thin sections (0.5 µm) were prepared and stained with Epoxy Tissue Stain (Electron Microscopy Sciences, Cat. # 14950) to screen samples with light microscopy. Ultrathin sections (70–80 nm) were generated from select blocks and mounted onto 100 mesh copper grids. Sections were stained with uranyl acetate (Ted Pella, Cat. # 19481) saturated in 50% ethanol for 30 min in the dark, washed in double distilled water, stained with Modified Sato’s lead stain [20] for 2 min, and washed again. Samples were viewed at the Fred Hutch Cancer Center, Seattle, WA, using a Thermo Fisher Talos L120 C transmission electron microscope with a Thermo Fisher Scientific Ceta CMOS high-resolution 16 M digital camera.

## 3. Results

### 3.1. Gross Pathology and Bacteriology

**Figure 1:**
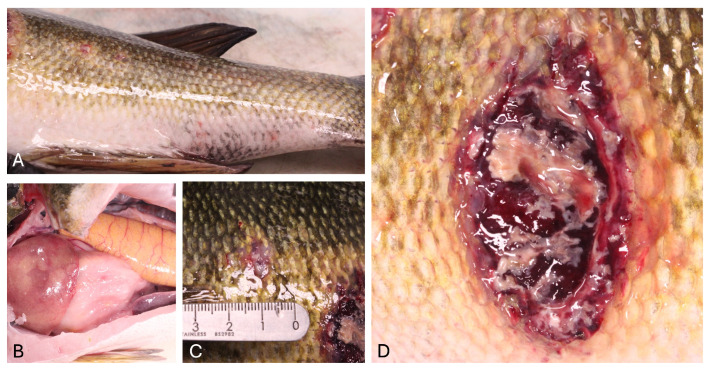
Gross lesions in the smallmouth bass (*Micropterus dolomieu*). (**A**) Cutaneous ulcers and erythema on the left flank and ventrum. (**B**) Liver with pale tan mottling, ovary, spleen, and coelomic adipose tissue. (**C**) Cutaneous ulcers on the left flank. (**D**) Large cutaneous ulcer on the left flank, measuring 7.0 cm × 4.0 cm, with central necrotic tissue and exposure of the underlying muscle.

Gross necropsy revealed multifocal skin ulcers ranging in diameter from 1.0–7.0 cm on the lateral body wall and ventral peduncle of the fish, caudal to the pectoral fins and below the lateral line. These lesions were characterized by an irregular margin of red, inflamed skin surrounding centrally necrotic, tan to yellow tissue with exposure of the underlying muscle (Figure 1). Wet mounts of skin and gills were unremarkable. Upon internal examination, the spleen was diffusely and moderately enlarged, the liver was mottled pale orange to yellow, and there was diffuse pallor of the posterior kidney. One colony of a gram-positive bacillus was isolated from a sample of kidney but was not able to be identified using traditional methods. No bacterial growth was observed for the sample of brain tissue.

### 3.2. Histopathology

Microscopic evaluation of the skin lesions revealed ulceration of the epidermis with extensive granulation tissue formation, multifocal inflammation by large numbers of macrophages, lymphocytes, neutrophils, and eosinophilic granular cells, and few granulomas, characterized by central necrotic debris rimmed by epithelioid macrophages. Inflammation extended from the epidermis and superficial dermis into the deep dermis and underlying skeletal muscle. No bacterial, fungal, or parasitic agents were seen in Grocott’s methenamine silver, Fite’s acid-fast, or Brown–Hopps tissue gram stains. Within the spleen and anterior and posterior kidneys, aggregates of necrotic lymphocytes mixed with scattered pyknotic to karyorrhectic debris were observed, consistent with lymphocytolysis, although autolysis was also considered as a possible alternative cause. Additional histologic findings were indicative of widespread, systemic inflammation, and included keratitis and orbital cellulitis, epicarditis, encephalitis, and interstitial branchitis (Figure S1). In contrast to the marked and predominantly histiocytic inflammation within the skin lesions, inflammation within the eye, heart, brain, and gills was mild and predominantly lymphocytic, with intermixing of small numbers of neutrophils and eosinophilic granular cells.

### 3.3. In Situ Hybridization

**Figure 2:**
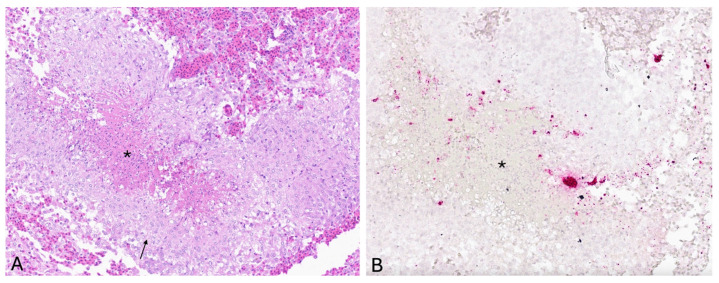
Matching H&E histopathology and tissue distribution of largemouth bass virus (LMBV) in a section of skin ulcer by in situ hybridization (ISH). (**A**) Granuloma within the skin, with central necrotic debris (asterisk) rimmed by epithelioid macrophages (arrow), H&E. (**B**) Multifocal positive reactivity for LMBV localized predominantly at the margin of epithelioid macrophages and central necrosis (asterisk).

Viral nucleic acid was visualized in sections of skin ulcer and spleen and was not detected in the kidneys, liver, heart, or brain. Infected cells were observed within granulomas in the dermis, localized predominantly at the margin of epithelioid macrophages and central necrosis (Figure 2). Multifocal, punctate positive signals were observed within the splenic lymphoid tissue, which were interpreted as positive detection of viral nucleic acid. Viral nucleic acid was not visualized in the kidneys, but there were occasional positive signals interpreted as large stain precipitates. Positive and negative control staining results were as expected on test tissues.

### 3.4. Virus Isolation and PCR Amplification

FHM cells incubated at 25 °C showed considerable CPE on the first passage 3 days post-inoculation, characterized by enlarged, refractile, and lysed cells detaching from the monolayer with destruction of more than 90% of the monolayer at the 1:10 dilution, more than 50% of the monolayer at the 1:100 dilution, and more than 10% of the monolayer of the 1:1000 dilution.

CPE was not observed on EPC, CHSE-214, BF-2, and FHM cell lines incubated at 15 °C for either the primary inoculation or first passage, which totaled a 28-day incubation period.

LMBV was detected by conventional PCR followed by Sanger sequencing from a homogenized skin sample and FFPE skin sample. The consensus sequence was identical (246/246 bp) to seven LMBV strains isolated from largemouth and smallmouth bass in North America (e.g., GenBank accession no. PP526145.1). LMBV was not detected by conventional PCR in a FFPE spleen sample.

### 3.5. Whole-Genome Assembly and Phylogenetic and Genetic Analyses

**Figure 3:**
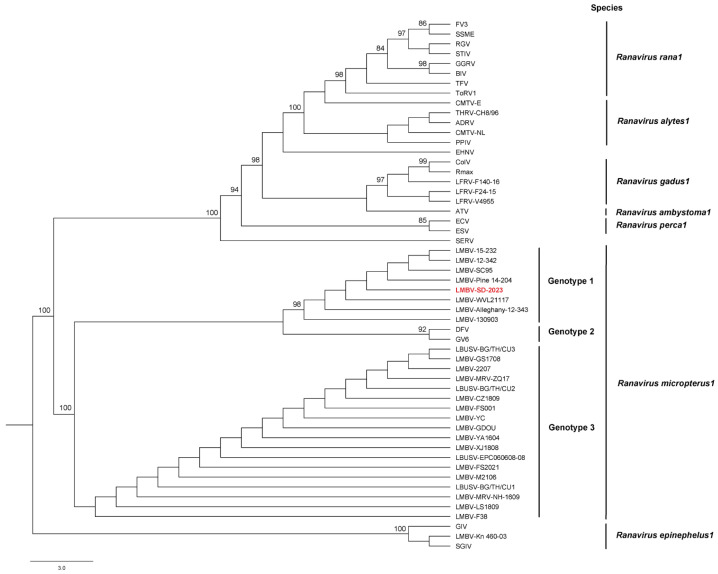
Maximum likelihood cladogram depicting the relationship of the largemouth bass virus isolate (SD-2023) (red) to 53 ranaviruses, based on the nucleotide sequence alignment of the major capsid protein gene. Numbers at each node represent bootstrap support of the ML analysis (values ≥ 80% shown). See Table S1 for viral taxa abbreviations.

The NGS yielded 13,262,492 paired-end reads for the LMBV-SD-2023 isolate. The trimming and host removal steps yielded 5,449,693 paired-end reads that were assembled and generated a large contig (99,179 bp; 51.9% G + C). Alignment of the 5,449,693 paired-end reads to this contig incorporated 97.7% of reads at an average coverage depth of 13,723 reads/nt. The contig was determined to be the complete genome of the LMBV isolate (SD-2023) by examining reads that spanned both the sequence beginning and end. BLASTN analysis of the LMBV isolate (SD-2023) genome revealed the top two matches (>99.8% identity; 100% coverage) were matched to complete genome sequences of LMBV isolates recovered from largemouth bass (LMBV strain Pine 14–204, GenBank accession no. MK681856.1 [2], and LMBV-WVL21117, GenBank accession no. PP526145 [6]) in North America. The genome of the LMBV isolate (SD-2023) was predicted to encode 100 ORFs (Figure S2). The ML analysis based on the MCP gene alignment generated a relatively resolved tree (Figure 3). Similar to the BLASTN analysis, the LMBV isolate (SD-2023) was supported as a member of the species *Ranavirus micropterus1*. The LMBV isolate (SD-2023) branched within a clade of LMBV isolates recovered from North American largemouth or smallmouth bass (Figure 3, Table S1). While the phylogenetic analysis strongly supported the monophyly of genotypes 1 and 2 (bootstrap values of 98 and 92, respectively), it did not support the monophyly of genotype 3 (bootstrap value of 0). There was moderate support (bootstrap value of 77) for genotype 1 and 2 as sister clades.

### 3.6. Electron Microscopy

**Figure 4:**
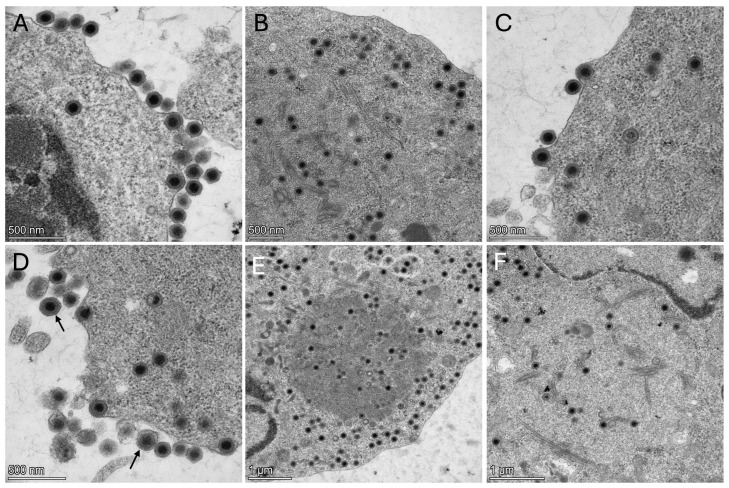
Largemouth bass virus in fathead minnow (*Pimephales promelas*) cell line 48 h after infection. (**A**–**C**) Non-enveloped virions in the cell cytoplasm with an icosahedral capsid containing an electron-dense core. Notice viral budding in (**A**,**C**). (**D**) Non-enveloped virions budding from the cell membrane, with few virions enveloped by the cellular plasma membrane (arrows). (**E**,**F**) Viral assembly regions within the cell cytoplasm demarcated by an area of electron dense (**E**) or electron lucent (**F**) cytoplasm containing cross and longitudinal sections of tubular elements.

Mature viral particles in the cell cytoplasm were non-enveloped with an icosahedral capsid containing an electron-dense core. Virus diameter averaged 134 nm (range = 123–148 nm, standard deviation = 8.2 nm) based on 10 measurements. Most extracellular virus particles were non-enveloped, though few virus particles were enveloped by the cellular plasma membrane as a result of virus budding from the cell. Viral assembly regions within the cell cytoplasm were demarcated by an area of electron lucent or electron dense cytoplasm containing cross and longitudinal sections of tubular elements, while devoid of other cellular organelles (Figure 4). Heavily infected cells had evidence of degeneration, including nuclei with condensed chromatin, loss of nuclear membrane and structure, and cell lysis associated with disruption of the cellular plasma membrane.

## 4. Discussion

In this study, we report the detection, isolation, genomic characterization, and gross and histologic features of LMBV infection in a representative wild smallmouth bass from a larger outbreak of ulcerative skin lesions in Lake Oahe, together with localization of viral nucleic acid via in situ hybridization and visual analysis of viral particles using transmission electron microscopy.

The ML phylogenetic analysis based on the MCP gene alignment supported the LMBV isolate (SD-2023) as a member of the species *Ranavirus micropterus1* (Figure 3). Among the 28 LMBV MCP gene sequences available for inclusion in the ML analysis, the LMBV isolate (SD-2023) branched within the subclade of LMBV isolates recovered from North American largemouth and smallmouth bass (genotype 1). The two other genotypes include viral sequences generated from infected largemouth bass and other cultured and wild fish species in Asia [6] and Australia [21]. To the authors’ knowledge, this is the first detection of LMBV in wild smallmouth bass from South Dakota. Previous isolations of LMBV from wild smallmouth bass displaying ulcerative skin lesions or as part of fish-kills have occurred in the Susquehanna and Allegheny river systems [2], the Green Bay waters of Lake Michigan [6], and the Chesapeake Bay watershed [22]. Detection of LMBV from smallmouth bass have occurred in the following states: AZ, KY, MI, MN, NY, OH, OK, PA, SD, WI, WV [3].

Gross and histologic features in this case were consistent with previously reported pathologic features in LMBV-infected fish [23], including severe ulcerative skin lesions (the most consistent finding in LMBV infection of smallmouth bass in some reports [6]) and associated myositis, along with lymphocytolysis within the spleen, anterior, and posterior kidneys. Keratitis and orbital cellulitis, epicarditis, encephalitis, and interstitial branchitis were also noted in this case, indicative of widespread, systemic inflammation, and are less commonly reported findings in LMBV-infected fish. Other lesions seen in LMBV infection in smallmouth bass include necrotizing steatitis and coelomitis, internal and external hemorrhages, and hepatic and splenic necrosis [2]. Interestingly, in an experimental study, smallmouth bass infected with LMBV via immersion developed ulcerative dermatitis and exophthalmia due to necrotizing panuveitis and keratitis, while those infected via intracoelomic injection did not [2]. The presence of keratitis and orbital cellulitis in the current case are supportive of a route of infection most similar to experimental immersion. Although intracoelomic injection is not a natural method of infection, this variation of lesions based on route of infection suggests that LMBV has tropism for cell types of different origins and that different routes of infection are effective for transmitting disease.

Experimental infection studies have largely been performed using largemouth bass, in which LMBV has been shown to infect almost all host tissues and organs, including the gill, swim bladder, anterior and posterior kidney, liver, cecum, gonad, brain, spleen, heart, and intestine, with variation in viral load in different organs depending on the route of LMBV infection. In largemouth bass infected via immersion, the gills, swim bladder, and posterior kidneys were the most frequently infected organs [24], suggesting that the gills may represent an important portal of entry. Following intracoelomic injection, viral load reached its peak at day 7 post infection, with the highest viral loads in the spleen, followed by liver, intestine, and kidney [25], while oral infection via gavage resulted in the highest viral titers in the swim bladder, anterior kidney, and cutaneous mucus [26]. Detection of LMBV in the cutaneous mucus of infected fish suggests the potential involvement of the skin as a pathway for LMBV invasion and viral shedding.

In the current study, LMBV viral nucleic acid was localized via ISH within histologic lesions in the skin. Viral nucleic acid was concentrated within granulomas in the dermis, predominantly at the margin of epithelioid macrophages and central necrosis. These cellular localization findings suggest an important role for macrophages in sequestering LMBV within granulomas, which are histologically distinct, organized structures formed by aggregates of macrophages in response to persistent stimuli to contain and isolate the offending agent and to establish a localized immune response. Following visualization of viral nucleic acid via ISH in histologic sections of skin ulcer, PCR was subsequently performed on the same block of formalin fixed, paraffin embedded skin tissue, and LMBV was detected, providing further support for cutaneous virus localization. Multifocal punctate positive signals were also observed within splenic lymphoid tissue, interpreted as positive detection of viral nucleic acid; however, LMBV was not detected via subsequent PCR performed on the same block of formalin-fixed, paraffin-embedded spleen tissue. These discordant results may either indicate a false positive ISH result or a false negative PCR result, possibly because the total viral DNA in the spleen sample was below the limit of detection of the PCR assay.

The current study represents the first report to use ISH as a tool to characterize natural LMBV infection. Documentation of the systemic distribution of the LMBV nucleic acid in infected tissues strongly supports the usefulness of ISH in characterizing viral infection and in confirming its cellular location in natural infection. The visualization of viral nucleic acid by ISH in infected tissues corresponding to histologic lesions powerfully complements viral detection via PCR, and the localization can indicate possible disease pathogeneses. The only previous report to use ISH to characterize LMBV infection used whole-mount in situ hybridization (WISH) to detect LMBV in zebrafish (*Danio rerio*) embryos and larvae after experimental brain infection, first observing positive signals in the brain that increased from 12 h post injection, with signal detected within caudal hematopoietic tissue at 72 h post infection. This zebrafish study also utilized electron microscopy to visualize icosahedral-shaped viral particles within infected mesenchymal stromal cells and endothelial cells, which sometimes displayed destruction of mitochondria and loss of cristae; no viral particles were detected in skeletal muscle cells in infected larvae [27].

In vivo and in vitro experimental studies have indicated that water temperature plays a role in the susceptibility of fish to LMBV infection. Largemouth bass injected with LMBV at 30 °C experienced significantly higher mortality rates and higher viral loads than those injected at 25 °C [28]. Similarly, smallmouth bass exposed to LMBV at 28 °C experienced 50% mortality as compared to 10% and zero mortality when exposed to LMBV at 23 °C and 11 °C, respectively. This increased susceptibility may reflect alterations in host immune competency, LMBV virulence, or both. Previous in vitro studies indicate that higher temperatures favor LMBV replication, and that the optimal incubation temperature for LMBV replication is 30 °C [29]. These findings correlate with virus isolation in the current case, in which in vitro virus amplification occurred at 25 °C but not at 15 °C. Additionally, higher water temperatures may be associated with other stressors for fish, such as low dissolved oxygen concentrations, which may contribute to host defense mechanism compromise.

In the current study, the diseased smallmouth bass from Lake Oahe were reported during the months of September and October, following a spike in fishing pressure and during high ambient temperatures. Average temperatures in the region, based on National Weather Service Data from the Pierre Regional Airport, were 74.4 °F (23.6 °C) in August and 66.2 °F (19.0 °C) in September, respectively 1.4 and 2.6 °F above the historical average temperatures for these months [30]. A gillnetting survey that had been performed in September by the South Dakota Department of Game, Fish and Parks may also have been a contributing stressor. The general importance of the environment for LMBV pathogenicity is supported by scrutiny of available environmental data from local epidemics: a mortality event of approximately 3000 largemouth bass at the Sardis Reservoir in Mississippi in September 1998 was precipitated by high water temperatures of 29 to 32 °C, high fishing pressure, and destratification leading to low dissolved oxygen [31]. Subsequent surveys at the Sardis Reservoir during the year following the outbreak revealed long-term persistence of LMBV in 30–50% of sampled fish, but with significantly less severe clinical disease compared to in September, suggesting that environmental factors, including warmer water temperatures, contributed to the development of clinical disease in this outbreak. Fish with a higher thermal tolerance and/or preference for warmer water temperatures, such as juvenile smallmouth and largemouth bass, may therefore be uniquely vulnerable to developing clinical disease following LMBV infection [32]; a vulnerability likely to be intensified by climate change and increasing global water temperatures.

Further sampling would be needed to determine the geographic distribution, affected populations, and evolutionary relationship between isolates of LMBV. Factors including management, coinfections, environmental stressors, and viral and host genetics may contribute to virulence and pathology of LMBV. Further studies could elucidate these contributing factors and to bridge our current knowledge gaps in our understanding of the viral pathogenesis of LMBV.

## Data Availability

The complete genome sequence of the largemouth bass virus isolate SD-2023 has been deposited in GenBank under accession no. PV833272.

## Supplementary Materials

The following supporting information can be downloaded at: https://www.mdpi.com/article/10.3390/v17081031/s1, Figure S1: Histologic lesions in the smallmouth bass.; Table S1: Virus species, isolate name, abbreviations, and GenBank accession numbers of the ranaviruses used in the phylogenetic analyses.; Figure S2: Genome map for largemouth bass virus isolate SD-2023.

## Abbreviations

The following abbreviations are used in this manuscript:

BF-2	Bluegill fry cell line
°C	Degrees Celsius
CHSE-214	Chinook salmon embryo cell line
°F	Degrees Fahrenheit
CPE	Cytopathic effects
EPC	*Epithelioma papulosum cyprini* cell line
FFPE	Formalin fixed paraffin embedded
FHM	Fathead minnow cell line
H&E	Hematoxylin and eosin
ISH	In situ hybridization
LMBV	Largemouth bass virus
MCP	Major capsid protein
ML	Maximum likelihood
NGS	Next generation sequencing
No.	Number
PCR	Polymerase chain reaction
RNA	Ribonucleic acid
WISH	Whole-mount in situ hybridization
