# Supplementary material for: Pathology, Tissue Distribution, and Phylogenetic Characterization of Largemouth Bass Virus Isolated from a Wild Smallmouth Bass (Micropterus dolomieu)
Source: Viruses. 2025 Jul 23;17(8):1031. doi: 10.3390/v17081031 (PMC12390719; doi:10.3390/v17081031)
Supplement: Supplementary file 1 [file viruses-17-01031-s001.zip › Supplemental Figures.pdf]

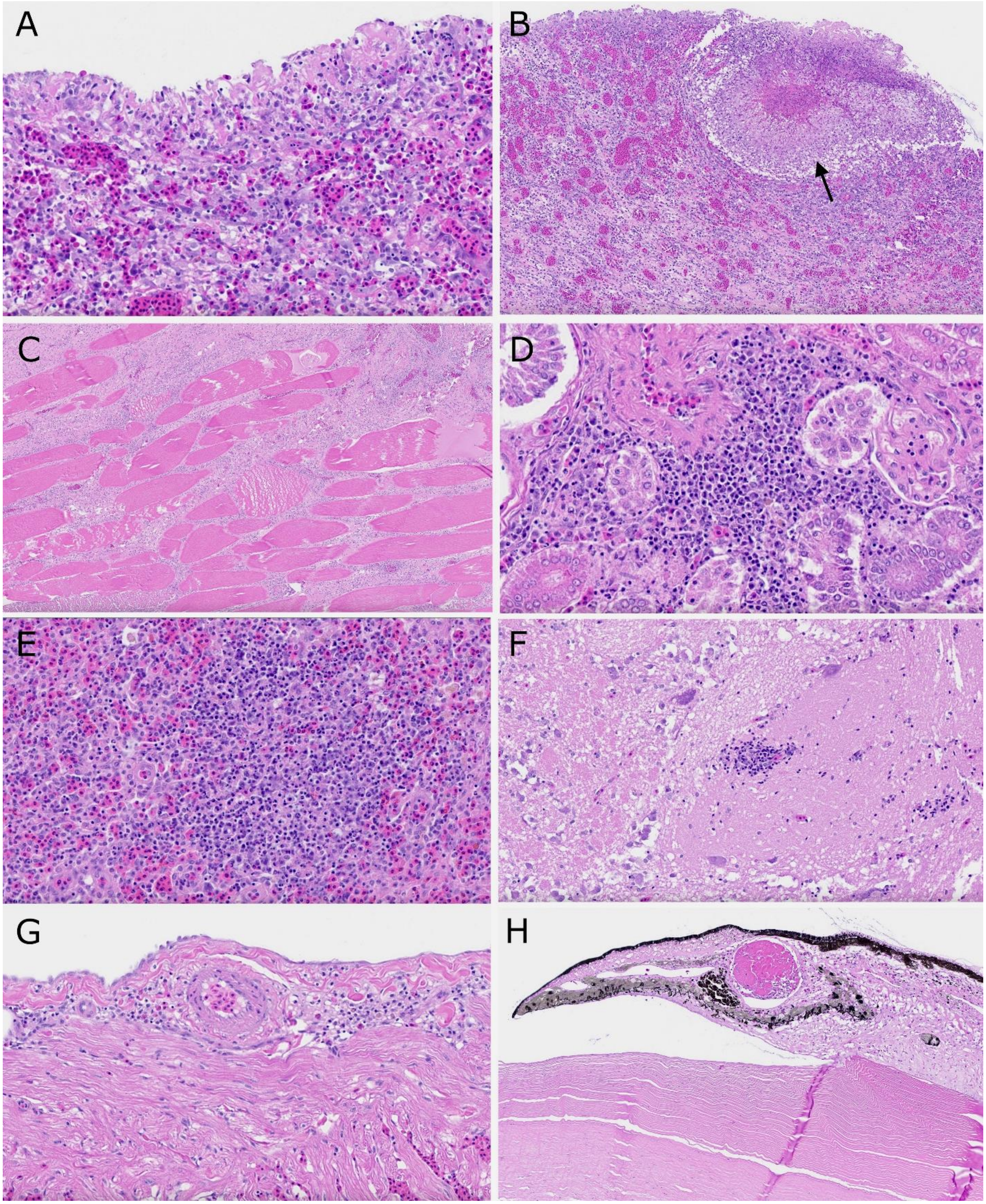

Figure S1. Histologic lesions in smallmouth bass (*Micropterus dolomieu*). (A and B) Ulcerative dermatitis with extensive granulation tissue formation, inflammatory infiltration by large numbers of macrophages, lymphocytes, neutrophils, and eosinophilic granular cells, and granuloma formation (arrow). (C)

Granulomatous myositis and degeneration of skeletal muscle subjacent to the cutaneous ulcer. (D) Necrosis of lymphocytes (lymphocytolysis) within the lymphoid tissue of posterior kidney. (E) Necrosis of lymphocytes (lymphocytolysis) within the lymphoid tissue of the spleen. (F) Multifocal, moderate lymphocytic encephalitis. (G) Multifocal, mild, lymphocytic epicarditis. (H) Iridal thrombus within the eye.

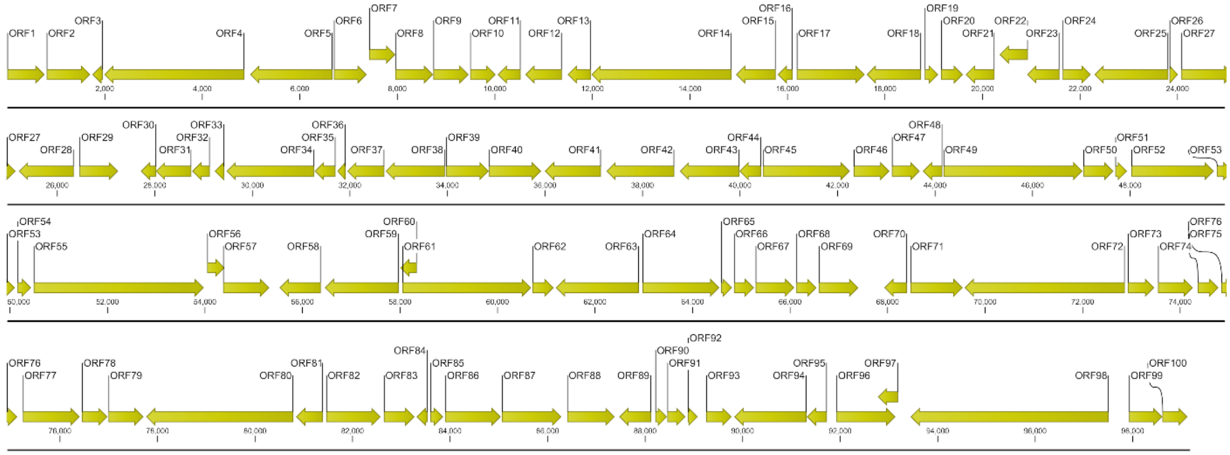

Figure S2. Genome map for largemouth bass virus isolate SD-2023. The 100 predicted functional open reading frames are indicated by yellow arrows.
