# Supplementary material for: Pathology, Tissue Distribution, and Phylogenetic Characterization of Largemouth Bass Virus Isolated from a Wild Smallmouth Bass (Micropterus dolomieu)
Source: Viruses. 2025 Jul 23;17(8):1031. doi: 10.3390/v17081031 (PMC12390719; doi:10.3390/v17081031)
Supplement: Supplementary file 1 [file viruses-17-01031-s001.zip › Table S1.pdf]

Table S1. Virus species, isolate name, abbreviations, and GenBank accession numbers of the ranaviruses used in the phylogenetic analyses.

Virus species	Isolate name (abbreviation)	GenBank accession number
Ranavirus rana1	Frog virus 3 (FV3)	AY548484
	Frog virus 3 isolate SSME (SSME)	KJ175144
	Soft-shelled turtle iridovirus (STIV)	EU627010
	Rana grylio iridovirus (RGV)	JQ654586
	Tiger frog virus (TFV-China)	AF389451
	Tortoise ranavirus isolate 1 (ToRV1)	KP266743
	Bohle iridovirus (BIV)	KX185156
	German gecko ranavirus (GGRV)	KP266742
Ranavirus ambystoma1	Ambystoma tigrinum virus (ATV)	AY150217
Ranavirus perca1	Epizootic haematopoietic necrosis virus (EHNV)	FJ433873
	European catfish virus (ECV)	KT989885
	European sheatfish virus (ESV)	JQ724856
Ranavirus alytes1	Common midwife toad virus (CMTV-E)	JQ231222
	Common midwife toad virus (CMTV-NL)	KP056312
	Testudo hermanni ranavirus (THRV-CH8/96)	KP266741
	Andrias davidianus ranavirus (ADRV)	KC865735
	Pike-perch iridovirus (PPIV)	KX574341
Ranavirus gadus1	Lumpfish ranavirus isolate F140-16 (LFRV-F140-16)	MH665359
	Lumpfish ranavirus isolate F24-15 (LFRV-F24-15)	MH665358
	Lumpfish ranavirus isolate V4955 (LFRV-V4955)	MH665360
	Ranavirus maximus (Rmax)	KX574343
	Cod iridovirus (CoIV)	KX574342
Ranavirus micropterus1	Largemouth bass virus isolate Alleghany 12-343 (LMBV-Alleghany 12-343)	MK681855
	Largemouth bass virus isolate Pine 14-204 (LMBV-Pine 14-204)	MK681856
	Largemouth bass virus isolate LMBV-FS001 (LMBV-FS001)	ON936874
	Mandarin fish ranavirus strain NH-1609 (LMBV-MRV_NH-1609)	MG941005
	Largemouth bass virus isolate WVL21117 (LMBV-WVL21117)	PP526145-1
	Micropterus salmoides ranavirus isolate M2106 (LMBV-M2106)	OQ267587
	Siniperca chuatsi ranavirus isolate 2207 (LMBV-2207)	OQ267588
	Largemouth bass virus strain GDOU (LMBV-GDOU)	MW630113
	Largemouth bass virus isolate Santee-Cooper Reservoir (LMBV- SC95)	FR682503
	Largemouth bass virus isolate 12-342 (LMBV-12-342)	KY825779
	Largemouth bass virus isolate LMBV-FS2021 (LMBV-FS2021)	ON418985
	Largemouth bass virus isolate LMBV-YC (LMBV-YC)	PV459226
	Santee-Cooper ranavirus isolate BG/TH/CU3 (LBUSV-BG/ TH/CU3)	KU507317
	Mandarin fish ranavirus isolate MRV-ZQ17 (LMBV-MRV-ZQ17)	PV200168
	Halichoeres melanurus ranavirus isolate F38 (LMBV-F38)	ON595973
	Largemouth bass ulcerative syndrome virus isolate EPC060608-08 (LBUSV- EPC060608-08)	GU256635
	Doctor fish virus (DFV)	FR677324
	Guppy virus 6 (GV6)	FR677325
	Largemouth bass virus isolate 15-232 (LMBV-15-232)	KY825781
	Largemouth bass virus isolate 130903 (LMBV-130903)	KY825782
	Largemouth bass virus isolate LS1809 (LMBV-LS1809)	MK836315

	Largemouth bass virus isolate CZ1809 (LMBV-CZ1809)	MK836316
	Largemouth bass virus isolate XJ1808 (LMBV-XJ1808)	MK836317
	Largemouth bass virus isolate GS1708 (LMBV-GS1708)	MK836318
	Largemouth bass virus isolate YA1604 (LMBV-YA1604)	MK836319
	Santee-Cooper ranavirus isolate BG/TH/CU1 (LBUSV-BG/TH/CU1)	KU507315
	Santee-Cooper ranavirus isolate BG/TH/CU1(LBUSV-BG/TH/CU2)	KU507316
	Largemouth bass virus isolate South Dakota 2023 (LMBV-SD-2023)	PV833272
Ranavirus epinephelus1	Singapore grouper iridovirus (SGIV)	AY521625
	Grouper iridovirus (GIV)	AY666015
	Largemouth bass virus isolate Kn 460-03 (LMBV-Kn 460-03)	JF264364
Unclassified	Short-finned eel ranavirus (SERV)	KX353311
